# Characterization of the Tumor-Microenvironment in Patient-Derived Cervix Xenografts (OCICx)

**DOI:** 10.3390/cancers4030821

**Published:** 2012-08-29

**Authors:** Naz Chaudary, Melania Pintilie, Joerg Schwock, Neesha Dhani, Blaise Clarke, Michael Milosevic, Anthony Fyles, Richard P. Hill

**Affiliations:** 1 Ontario Cancer Institute/Princess Margaret Cancer Centre and Campbell Family Institute for Cancer Research, University Health Network, Toronto, Ontario M5G 2M9, Canada; E-Mails: nchaudar@uhnres.utoronto.ca (N.C.); neesha.dhani@uhn.ca (N.D.); 2 Biostatistics Department, Ontario Cancer Institute/Princess Margaret Cancer Centre, University Health Network, Toronto, Ontario M5G 2M9, Canada; E-Mail: pintilie@uhnres.utoronto.ca; 3 Department of Laboratory Medicine and Pathobiology, University of Toronto, Toronto, Ontario M5G 2M9, Canada; E-Mails: joerg.schwock@gmail.com (J.S.); blaise.clarke@uhn.ca (B.C.); 4 Division of Medical Oncology and Hematology, Princess Margaret Cancer Centre, University Health Network, Toronto, Ontario M5G 2M9, Canada; 5 Department of Pathology, University Health Network, Toronto, Ontario M5G 2M9, Canada; 6 Department of Radiation Oncology, University of Toronto, Toronto, Ontario M5G 2M9, Canada; E-Mails: mike.milosevic@rmp.uhn.on.ca (M.M.); anthony.fyles@rmp.uhn.on.ca (A.F.); 7 Radiation Medicine Program, Princess Margaret Cancer Centre, University Health Network, Toronto, Ontario M5G 2M9, Canada; 8 Department of Medical Biophysics, University of Toronto, Toronto, Ontario M5G 2M9, Canada

**Keywords:** cervix cancer, stroma, patient-derived cervix xenograft, hypoxia, tumor microenvironment

## Abstract

*Rationale*: The tumor microenvironment (TME) is heterogeneous including both malignant and host cell components as well as regions of hypoxia, elevated interstitial fluid pressure (IFP) and poor nutrient supply. The quantitative extent to which the microenvironmental properties of primary tumors are recapitulated in xenograft models is not well characterized. *Methods*: Xenografts were generated by implanting tumor biopsies directly into the cervix of mice to create a panel of orthotopically-passaged xenografts (OCICx). Tumors were grown to ~1 cm (diameter) and IFP measurements recorded prior to sacrifice. Enlarged para-aortic lymph nodes (>1–2 mm) were excised for histologic confirmation of metastatic disease. Quantitative histological analysis was used to evaluate hypoxia, proliferation, lymphatic and blood vessels in the epithelial and stromal regions of the xenografts and original patient tumour. *Results*: IFP and nodal disease were not correlated with tumor engraftment. IFP measurements in the xenografts were generally lower than those in the patient’s tumor. Lymphatic metastasis increased with passage number as did levels of hypoxia in the epithelial component of the xenografts. The blood vessel density in the stromal component of the xenografts increased in parallel. When all the markers were compared between the biopsy and the respective 3rd generation xenograft 10 of 11 tumors showed a good correlation. *Conclusions*: This ongoing study provides characterization about tumoral and stromal heterogeneity in a unique orthotopic xenograft model.

## 1. Introduction

### 1.1. Cervical Cancer: Epidemiology and Clinical Challenges

Cancer of the uterine cervix represents the third most common cause of female mortality due to cancer worldwide [1,2], with an estimated 500,000 new cases diagnosed annually [3,4]. The mortality rates of this disease have remained relatively stable in economically developed countries with adequate screening practices, however morbidity and mortality remain high in countries with more limited infrastructure, where the implementation of screening and treatment strategies for early stage disease is challenging [2,5,6]. Epidemiologic studies have demonstrated a critical role of human papillomavirus (HPV) in the pathogenesis of neoplastic cervical lesions, with the “high risk” genotypes HPV 16/18 being associated with 99.7% of invasive cervical carcinomas [5,7,8]. Recently developed vaccines targeting oncogenic strains of HPV have demonstrated efficacy at reducing the incidence of HPV-related disease including both *in situ* and invasive cervix cancer. However, given the latency of HPV-driven cervix disease it is unlikely that these strategies will have a significant impact on the burden of cervix cancer for some time. It is therefore imperative to continue developing appropriate animal models of human cervix cancer, to facilitate an improved understanding of relevant tumor biology and for the identification and evaluation of novel therapies.

Cervical carcinomas develop in a stepwise manner from dysplastic lesions to carcinoma *in situ* and ultimately invasive cancer. Pathologically the majority, close to 85%, are squamous cell carcinomas (SCC) with the remainder being adeno-(AD) or adenosquamous carcinomas [9]. The success of screening strategies in reducing the morbidity and mortality related to cervical cancer is related to the effective management of early lesions by laser ablation, cryotherapy, or surgical excision, thereby impeding progression to invasive disease. [10,11,12]. Once established, invasive cancer is best managed either surgically or with radical radiotherapy depending on extent of tumor invasion [13]. Radical radiotherapy generally administered concurrently with cisplatin chemotherapy, is the preferred treatment option for locally advanced cervix cancer and results in 5-year disease-free survival rates of over 60% [14,15]. If disease recurrence occurs the patients, together with patients with diffuse metastatic disease at initial presentation, will then require systemic therapy, which is predominantly palliative in nature. These patients have a poor prognosis, as responses to chemotherapy are transient and patients continue to suffer significant morbidity related to pain and gastrointestinal or genitourinary obstruction, and eventually die of their disease [16,17].

### 1.2. Tumor Microenvironment

The profound cellular complexity of human cancers is now well established. Malignant epithelial cells share a physical space with a heterogeneous cellular stroma consisting of activated fibroblasts, immunomodulatory cells and vascular elements. In addition, heterotypic interactions amongst the different cellular components lead to deposition of a varied extracellular matrix of collagens and other proteins and polysaccharides, which provides a scaffolding structure for malignant cells [18,19]. Classically felt to be relevant in providing support to the malignant epithelial cells, it is now recognized that the different stromal components are also of critical significance in tumorigenesis and metastatic dissemination [20,21]. This is partially related to modifications epithelial tumor cells can initiate within their adjacent stroma creating a permissive and supportive (micro)environment facilitating their own growth and consequently tumor progression. Further, epithelial-stromal molecular cross-talk has significant implications for suppression of immuno-surveillance leading to immuno-tolerance of tumors [22]. Given the already described contributions of stroma biology to tumor progression and treatment resistance, investigations that further elucidate these interactions are especially relevant at this time [23,24].

The tumor microenvironment also exhibits abnormal pathophysiological features such as hypoxia and increased interstitial fluid pressure (IFP) associated with disorganized vascular development. Hypoxia develops when oxygen consumption within a tumor exceeds supply, and its presence has been demonstrated in a number of human cancers utilizing a variety of measurement techniques. Direct measurements of oxygen (O_2_) using polarographic electrodes have revealed low pO_2_ values in most cervical cancers [11,25,26,27,28]. Although first demonstrated to be a significant contributor in radiation resistance, other studies have also found a prognostic relevance of hypoxia, correlating with more aggressive tumor biology and poor patient outcome in a variety of tumor types including cervix cancer [29]. Many hypoxic cancers also show a greater metastatic potential, which is consistent with the idea that the tumor microenvironment has a major effect on cancer biology [30].

Another component of the tumor microenvironment, IFP, is also elevated in most solid tumors in comparison with normal tissue [19,27,28,31]. High IFP values reflect poor lymphatic drainage in tumors, increased vascular leakiness and decreased interstitial permeability [31,32]. Increased tumor IFP has been demonstrated to predict for inferior survival after irradiation [33]. Tumors in the cervix are accessible for biopsy and for measurements such as those of tumor hypoxia and IFP, both of which can be predictive for treatment outcome, making it a useful clinical model for examining pathophysiologic aspects of the tumor microenvironment that may apply to other cancers [28].

### 1.3. Patient-Derived Xenografts

The evaluation of the molecular status of tumors in response to treatment is integral for the rapid and efficient development of rational targeted anti-cancer therapies. It is only through the careful evaluation of on-treatment changes and perhaps even more importantly, those occurring on progression, that we will be able to understand the different tumor contexts predictive of response. Unfortunately obtaining repeat tumor biopsies, although certainly feasible in some contexts, provides a number of logistic challenges to the care of patients on clinical trials. Further, there are some primary tumors where location precludes being able to safely obtain adequate and repeated biopsies. For these reasons, it is crucial to have at our disposal appropriate preclinical models that closely parallel the clinical context on as many levels as is possible in terms of biological behavior, malignant pathophysiology and response to treatment.

Most xenograft models are generated by subcutaneously implantation, as the accessibility of this site contributes to the relative ease of developing and testing novel agents in these models. However the microenvironment of subcutaneous murine models may not reflect that of the original tumor from which the xenograft was derived [34]. Recapitulation of the original tumor microenvironment has a greater likelihood of occurring in orthotopic models. Currently the most commonly used murine tumor models are based on implantation of relatively homogenous human cell lines, which unfortunately do not adequately represent the clinical characteristics of the disease particularly with regards to drug response and distant metastasis. Therefore, establishing patient-derived tumor tissue xenograft models may provide a more accurate reflection of the tumor microenvironment than tumor cell lines [35]. Studies in a variety of tumor types including renal cell, prostate, osteosarcoma, myxoid liposarcoma, brain and uveal melanoma have already demonstrated consistency between clinical tumors and patient-derived xenografts with respect to histological and genetic profiling [36,37,38,39,40,41].

The aim of our study was to establish and quantify features of the tumor microenvironment of patient-derived xenograft models of cervix cancer. We studied a range of microenvironmental parameters (hypoxia, IFP, proliferation, vasculature, lymphatics, epithelial and stromal markers) separately in the epithelial and stromal compartments in serial passages of orthotopically maintained primary cervix xenografts, relating these measurements to those made in the primary patient tumors and/or biopsies from which the xenografts were derived. These analyses were performed on four different xenografts for each of the first five passages and on all the xenografts at passage three.

## 2. Experimental Section

### 2.1. Patients and Tissue Samples

Approval for this study was obtained from the Research Ethics Board at the University Health Network. Eligible patients were those undergoing an examination under anesthesia (EUA) as part of their pre-treatment evaluation. Patients agreeing to participate and providing informed consent had punch biopsies taken during EUA. At the same time measurements of both pO_2_ and IFP were made as described previously [31]. For pO_2_ measurements track length (1.6 to 2.2 cm) was selected according to the size of the tumor as determined clinically and from an MRI scan. A step length of 0.7 mm (1 mm forward, 0.3 mm back) was used, and 20 to 30 measurements per needle track were obtained. Where feasible, measurements were obtained at five positions symmetrically spaced around the circumference of the tumor to minimize intratumor heterogeneity [11].

Biopsies for implantation into mice were immediately placed in ice-cold alpha MEM medium containing antibiotics and processed as outlined below. Additional cervix biopsies were placed in OCT Optimum Cutting Temperature (OCT) medium (Sakura Finetek, Torrance, CA, USA) and frozen immediately in liquid nitrogen for immunohistochemistry.

### 2.2. Establishing First-Passage Orthotopic Primary Xenograft

All animal experiments were performed according to protocols approved under the regulations of the Canadian Council on Animal Care. Fresh cervix biopsy reserved for establishing xenograft, was cut into 1–2 mm pieces and immersed in Matrigel (BD Biosciences, Mississauga, ON, Canada). The pieces were used for orthotopic implantation into the cervix of SCID and NOD/SCID mice as described previously [42,43]. Part of the biopsy used for transplantation was placed in formalin for histology. The xenografts which grew were passaged using pieces of tumor tissue implanted into the same site for up to five passages and pieces of tumour were frozen at the passages 1 to 5. Mice were monitored up to one year post-biopsy implant for tumor growth before sacrifice if no palpable tumor growth was detected.

### 2.3. Tumor Microenvironment Analysis—Xenograft Processing

Mice received intraperitoneal EF5, [2-(2-nitro-1*H*-imidazol-1-yl)-*N*-(2,2,3,3,3-pentafluoropropyl)acetamide], 4 hours prior to sacrifice to mark hypoxic cells [44]. The interstitial fluid pressure (IFP) in the tumors was measured using a wick-in-needle technique as described previously [19]. IFP measurements were taken at three to four different locations close to the centre of the tumor, and the mean value of these readings was taken to represent the tumor IFP. The tumors were excised (~10 mm diameter), weighed and pieces fixed in formalin or flash frozen in OCT for histology. Para-aortic lymphnodes were excised for histological assessment of metastasis if >1 mm in diameter as described previously [43]. Tumor samples were later sectioned for staining with hematoxylin and eosin (H&E), and IHC analysis of hypoxia (external marker, EF5; endogeneous marker, carbonic anhydrase-9 (CA-9), [45,46]), vascular density (CD31), lymphatic vessels (LYVE1), proliferation (Ki67), smooth muscle actin (SMA), cytokeratin (cocktail AE1/AE3) and collagen type IV. The following antibodies and dilutions were used for immunohistochemistry: Rabbit anti-human polyclonal CA-9 (Novus Biologicals, Oakville, ON, Canada, NB100-417) 1:2000; Mouse anti-EF5 (Non commercial, gift from Cam Koch) 1:250; rabbit anti-mouse polyclonal CD31 (Santa Cruz, Santa Cruz, CA, USA), Sc-1506-R) 1:1000; anti-mouse polyclonal LYVE1 (rabbit) from Novus Biologicals (NB600-1008) 1:200; anti-human Ki67 (rabbit) from Neomarkers (Fremont, California, USA, M-9106-S1) 1:500. AE1/AE3 and SMA were stained using the Ventana Benchmark and uVIEW DAB detection system. AE1/AE3 (Dako, Burlington, ON, Canada, M3515), 1:200. SMA from Dako (M0851) and used at 1:400. Collagen IV (anti mouse for xenografts) Cedarlane 50451 AP-1 pepsin digestion; 1/1000 overnight incubation. Collagen IV (anti human for biopsies) Dako M0785; Citrate heat induced retrieval; 1/100 one (1) hour incubation. The IHC markers were quantified by identifying regions of staining to determine the fraction of the tumor tissue staining positive. Spectrum Genie Aperio analysis [47] was used to delineate epithelial and stromal components of the tumors. Quantification of the IHC markers was done separately for these two regions.

### 2.4. Statistical Methods

Association between the ability to form xenograft and the categorical clinical factors was analyzed utilizing the Fisher exact test and the Cochran-Armitage test. Spearman correlation coefficients were calculated between the primary tumors markers and the xenograft markers for all parameters assessed at passage 3. To investigate the changes of the markers for the different passages linear mixed effect modeling was applied. The residuals were checked and a log transformation was applied when necessary to stabilize the variance. To assess the reliability between patient and xenograft models we have calculated the Intraclass Correlation Coefficient (ICC).

## 3. Results

The success rate in establishing primary cervical xenograft models in the orthotopic site from patient cervix biopsy tissue was 48%, with 16 of 33 implanted tumors growing in the animals and maintained for up to 5 generations. Failure of engraftment was defined at 12 months post implantation. Details of the tumors giving rise to xenografts are outlined in Supplementary Table 1 showing the number of tumor in each passage. On average it took 3–4 months for the first palpable xenograft to arise following orthotopic transplant. The majority of the patients were FIGO stage II–IIIA/B with squamous cell histology. Analysis of association with outcome was non-informative due to both small patient numbers and lack of sufficient follow-up for some patients to assess clinical outcomes.

No associations were found between the xenograft take rate and the clinical characteristics of the patients, specifically histology, nodal status and tumor size, although the number of patients was small (n = 34) (Table 1). Similarly, there was no apparent association between IFP, level of hypoxia (HP5) or HPV status of the primary tumor and xenografting success rate. Associations with clinical outcome were not assessed because of the small number of patients and the limited time of follow-up since completing treatment.

**Table 1:** Patient nodal status and xenograft growth. There was no correlation between the number of cervix patients with negative, equivocal and positive nodal status with the number of xenografts grafted (*p* = 0.78). The total represents the number of biopsies implanted and sub-number of those grafted successfully. The number in brackets shows the percentage.

Nodal Status	Number of Patients	Xenograft Growth/(%)
**Negative**	8	4/(50)
**≤8 mm**		
**Equivocal**	6	2/(33)
**>8 mm and equal to or <1 cm**		
**Positive**	19	10/(52)
**>1 cm**		
**Total**	33	16 (48)

**Figure 1:**
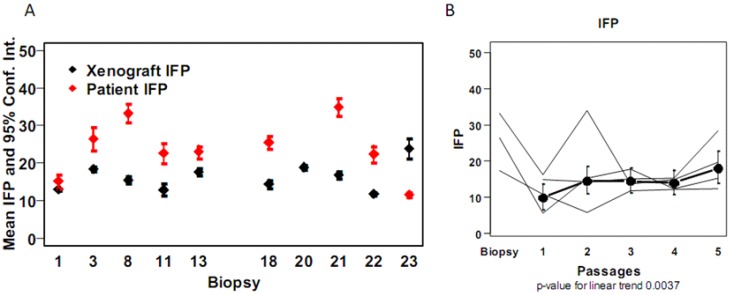
Interstitial fluid pressure levels in patient and the xenograft OCICx models. (**A**) IFP values are the mean ± SE mmHg with 3–4 measurements made for each tumor in all passages. Patient 16 did not have IFP measurements taken; (**B**) IFP (mmHg) measurements over passages for the OCICx 1, 3, 8, 16 are plotted with 95% confidence intervals at each passage individually and as a mean of the 4 models *p* value 0.0037.

Supplementary Table 1 illustrates our strategy for analysis of the various microenvironmental parameters of the xenografts. We opted to analyze four of the xenografts (OCICX 1, 3, 8, 16) at each of the first five passages and all of the xenografts at passage three for comparison with the initial biopsies from which the xenografts were established. The numbers in the highlighted regions of the body of the table represent the numbers of tumors that contributed to this analysis. Most of the patients had measurements of both IFP and hypoxia in their tumors hence initially we examined these parameters in the xenograft models. The analysis indicated similar but higher IFP values in most of the original patient tumors than in the passage three xenografts (Spearman correlation coefficient = 0.17) (Figure 1A). IFP increased slightly with the number of passages in the four xenograft models examined across five passages (*p* value for linear trend 0.0037). Unfortunately the value for one of the primary tumors was not available but it may be noted that for the other three values in the xenografts were generally lower than measured in the primary tumor (Figure 1B).

**Figure 2:**
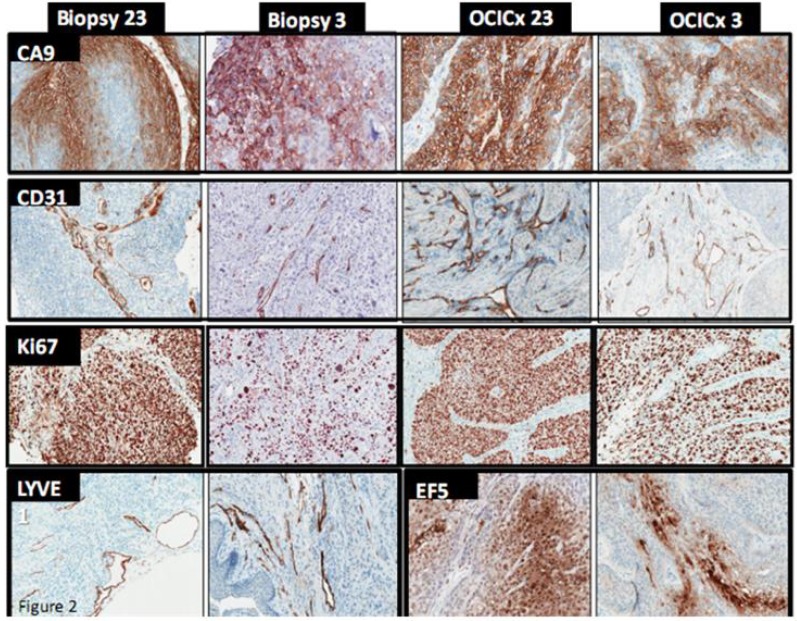
Hypoxia, blood vessel and proliferation staining. IHC staining in the Biopsy 23 and Biopsy 3 respectively for CA-9, EF5, CD31, Ki67 and LYVE1. 15× Magnification.

Measurements of hypoxia in the patients were made using an Eppendorf polarographic oxygen electrode (to determine the percent of pO_2_ values <5 mmHg = HP5) but this probe was not available for measurements in the xenografts. Consequently we used EF5 uptake in the xenografts and CA-9 staining in both the patient biopsies and the xenografts as surrogate parameters for tissue hypoxia (Figure 2). Both, EF5 and CA-9, were assessed separately in the stromal and tumor components of the tumor. EF5 had a moderate correlation with CA-9 expression in the xenografts (r = 0.51 in tumor and r = 0.52 in stroma), but the correlation between CA-9 and HP5 in the patients was poor. This poor correlation likely relates to the inherent heterogeneity of these different techniques in the assessment of tumoral hypoxia. We have reported previously that HP5 values made directly in the patient tumor do not correlate well with CA-9 measurements made in individual biopsies from the patient’s tumors [48]. (Figure 2).

**Figure 3:**
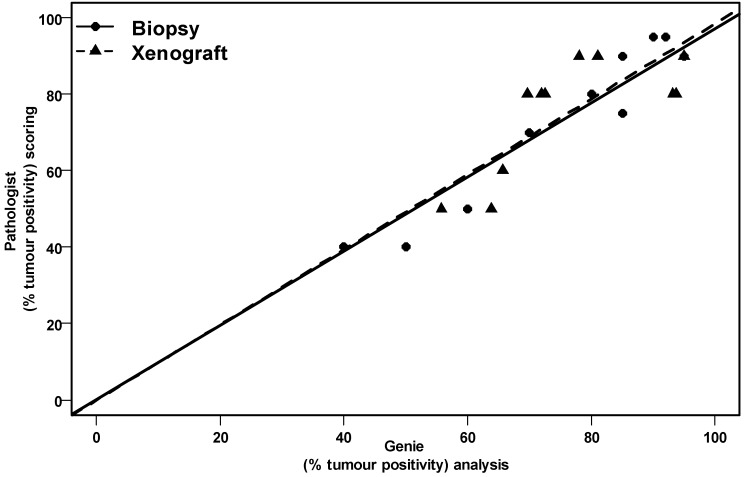
Linear plot regression analysis showing the association of percent tumor epithelial component from the pathologist scoring *vs.* Genie Spectrum software on patient cervix biopsies and xenograft. Scoring was conducted on the H&E slides with n = 1 slide for the biopsy (*p* = 0.0001) and n = 3–4 for the xenografts (*p* = 0.0099).

**Figure 4:**
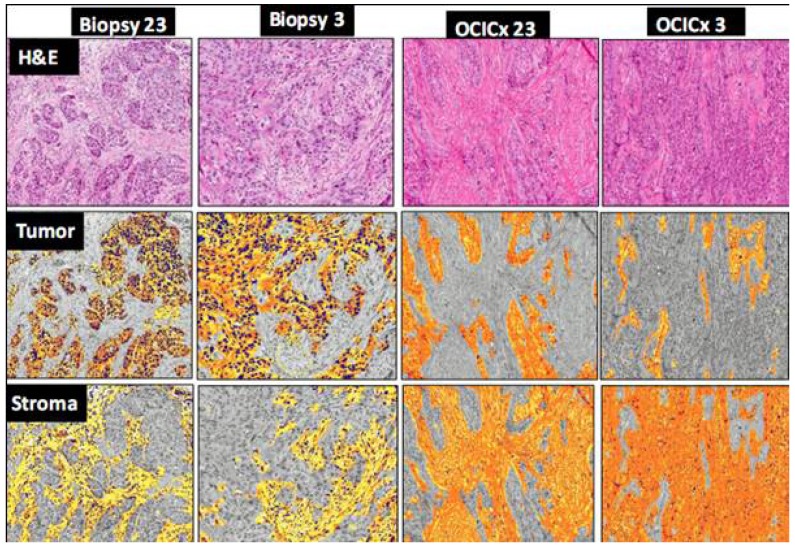
Genie images of tumor and stroma components in the biospy and OCICx. A panel of IHC staining for Biopsy 23 and Biopsy 3 showing H&E patient biopsy, positive pixel mask for tumor components; positive pixel mask for stroma components. Staining for OCICx 23 and OCICx 3 respectively H&E patient biopsy, positive pixel mask for tumor components; positive pixel mask for stroma components. 15× Magnification.

**Figure 5:**
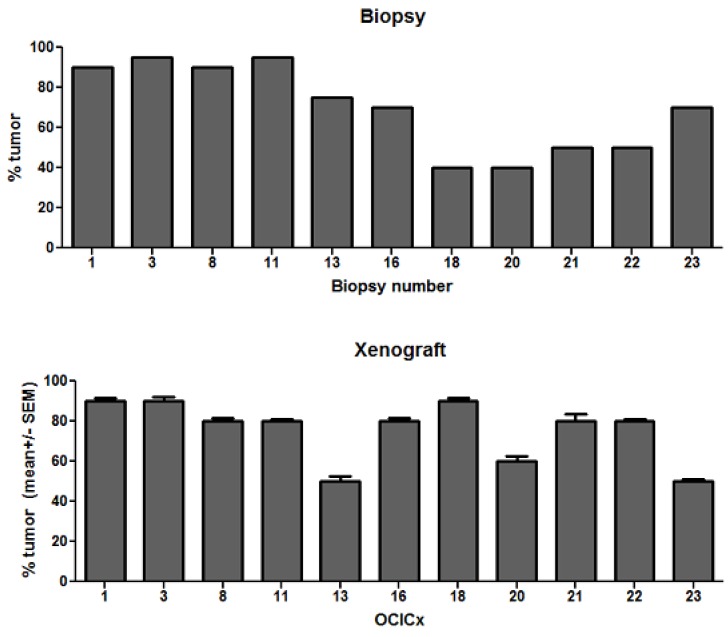
Percent tumor epithelial component of patient biopsy and OCICx at passage three for all models. Values plotted are mean ± SE n = 3 slides scored at passage three for the xenograft and n = 1 for patient biopsy.

The epithelial and stromal components of the original biopsies and xenograft models were scored and assessed using two independent methods—qualitatively by two pathologists (BC and JS) on high power fields and quantitatively using a semi-automated pattern recognition software program (Aperio’s Genie) on whole tissue sections. Scoring was done on the H&E slides on both the patient biopsy and xenograft sections for each OCICx model. Figure 3 shows a good accuracy of epithelial-stromal discrimination between these two approaches with a linear correlation for passage 3 xenografts and patient biopsies. The Genie Spectrum IHC analysis for the patient biopsy and matching (passage 3) xenograft is illustrated in Figure 4 for the tumor and stromal components of two of the models (OCICx 23 and 3). The panels illustrate the masks of tumor and stroma in the tumor sections with the corresponding H&E staining for comparison. The percent tumor at passage 3 for all the OCICx models and for the patient biopsy are shown in Figure 5. There is heterogeneity of tumor proportion between the two that probably reflects the fact that only a single (small) biopsy specimen was available, whereas values for the xenografts are averaged over a number of tumors (as indicated in Supplementary Table 1).

**Figure 6:**
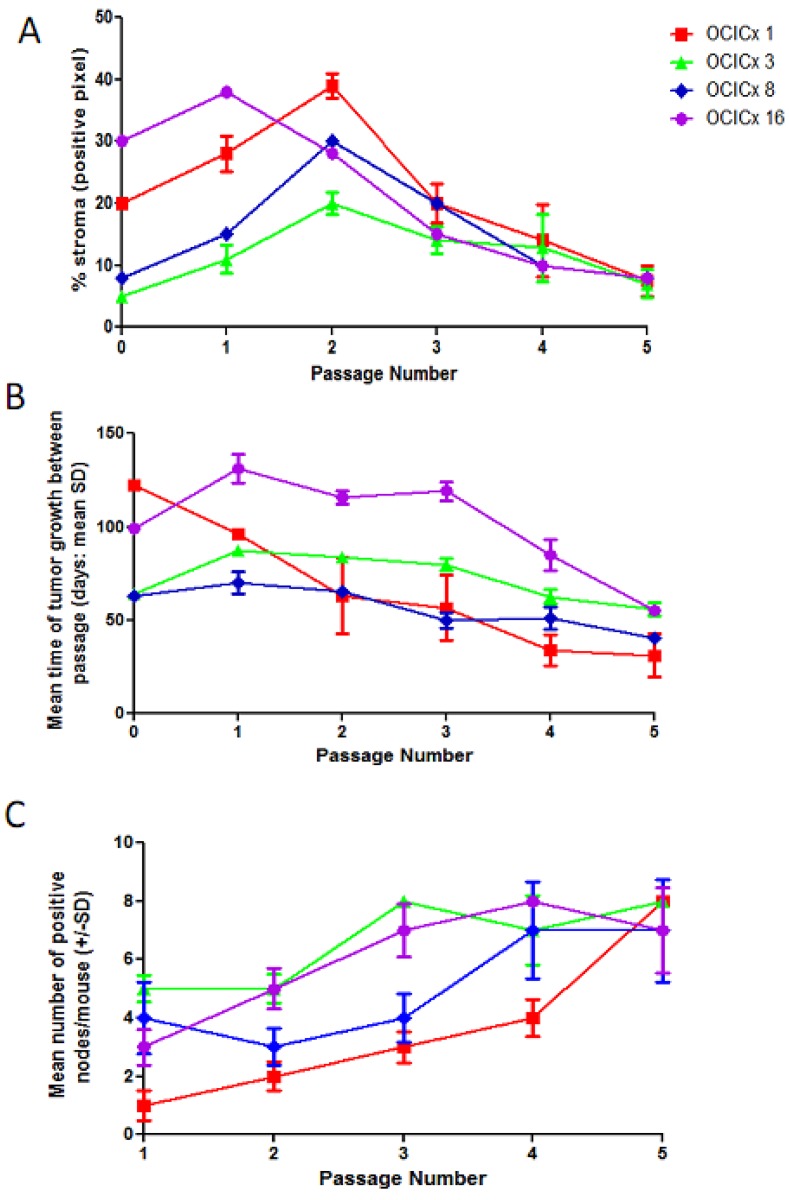
(**A**) Xenograft growth curves for the 4 OCICx models (1, 3, 8, 16) over passage. Tumor growth rates are plotted as function of passage number shown in days (mean time of all the xenografts in the passage ± SD); (**B**) The percent stroma as positive pixel counts over passage number generated by Genie Spectrum software based on the H&E analysis as mean ± SD. Passage number 0 indicates biopsy; (**C**) The mean number (±SD) of positive lymph nodes/mouse is shown for the 4 models over passage number.

**Supplementary Figure 1:**
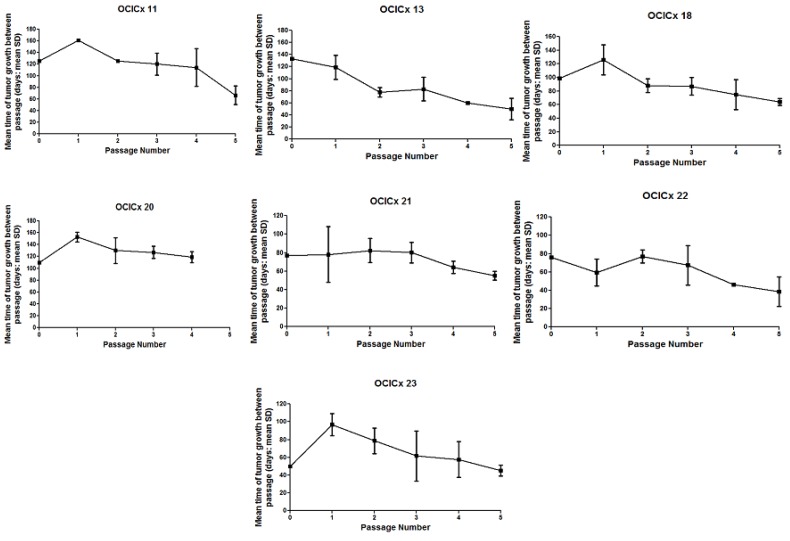
Mean time for tumor growth between passages for the OCICx models (11, 13, 18, 20, 21, 22, 23). Time in days are plotted in passage number shown (mean ± SD) for xenografts in that passage. Passage number at 0 indicates biopsy.

Using Genie analysis we quantified the stromal content of four xenografts models (OCICx 1,3,8, 16) throughout the five passages for comparison to the original biopsy (Figure 6A). The stromal components of the various passages relative to the biopsy from which they were derived shows heterogeneity (as seen in Figure 5). Interestingly, in all four models the level of stroma tended to increase at early passages and then decreased at later passages with a low value after five passages for all models (<10%). The decrease in stromal content in the later passages paralleled an increase in the growth rate of the tumors, as assessed by the mean time between passages (Figure 6B). This increase in growth rate was also seen in the remaining models (Supplementary Figure 1). Analysis of the para-aortic lymphnodes indicated that the metastatic potential of the xenografts also increased significantly (*p* < 0.01) with passage number (Figure 6C).

**Supplementary Figure 2:**
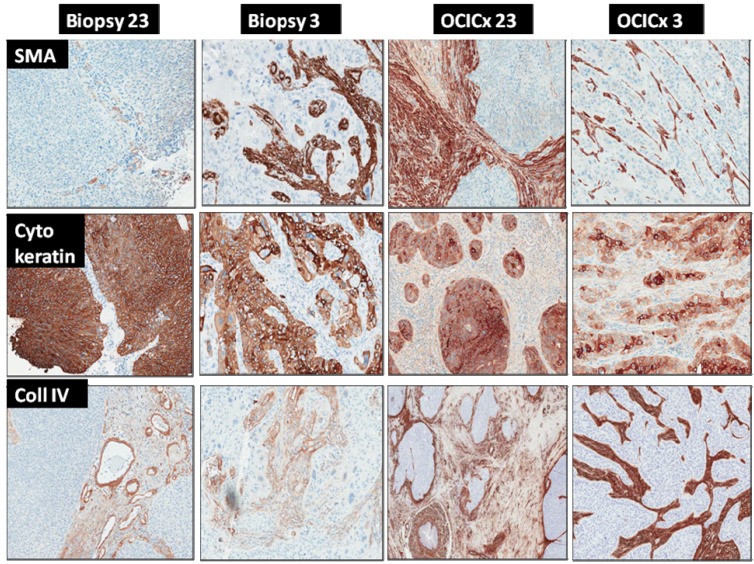
Immunohistochemical staining of stromal and epithelial components in the biopsy and OCICx xenografts. IHC staining in the Biopsy 23 and Biopsy 3 respectively smooth muscle actin (SMA); collagen IV (coll IV); AE1AE3 cytokeratin in parallel sections. 15× Magnification.

To gain better insight into the stromal and tumor epithelial components of the cervix xenografts and the associated cervix biopsy, SMA, collagen IV and cytokeratin staining were examined (Supplementary Figure 2). The distinct staining of SMA in the stromal components showed well-differentiated fibroblasts/myofibroblasts. Collagen IV staining was also very specific for the stroma. The cytokeratin IHC stain in both the biopsy and xenograft differentiates (positive) epithelial components from (negative) stroma. Proliferation (Ki-67 staining), hypoxia (EF5 and CA-9 staining), and blood vessels (CD31 staining) and lymph vessels (LYVE1 staining) were also assessed in these biopsies and xenografts as shown in Figure 2.

**Figure 7:**
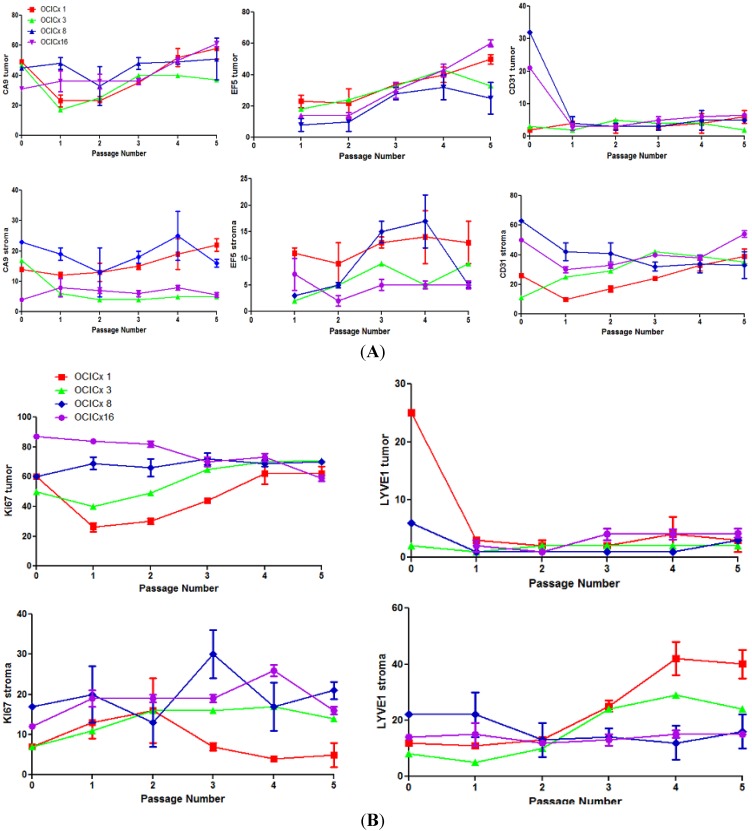
Quantitation of the staining for the various markers in the tumor and stromal components separately in the 4 models (OCICx 1, 3, 8, 16) over passage number. The percent pixel positivity is shown for each marker (**A**) CA-9, EF5, CD31; (**B**) Ki67 and LYVE1. Values plotted are mean ± SD. Passage 0 indicates biopsy piece.

To alleviate issues associated with heterogeneity in the tumor to stromal content of the biopsies and xenografts, quantitation of the staining for the various markers was assessed separately in the tumor and stromal components as shown in Figure 7A,B. For CA-9 and EF5 (hypoxia) in the epithelial components of the tumor there was a significant (*p* < 0.001) increase with passage number. In parallel there was a significant increase in vascular staining (CD31) (*p* < 0.001) in the stromal component. On average there was also a significant increase in Ki67 staining in the epithelial component of the tumor and LYVE1 in both components, however this is variable from one model to another. CD31 and LYVE1 levels were much lower in the epithelial than in the stromal component consistent with the finding that CA-9 and EF5 change to a larger extent in the epithelial than the stromal component. Ki-67 levels are much lower in the stromal than epithelial component. Correlations between these markers were in general weak (r < 0.6).

**Figure 8:**
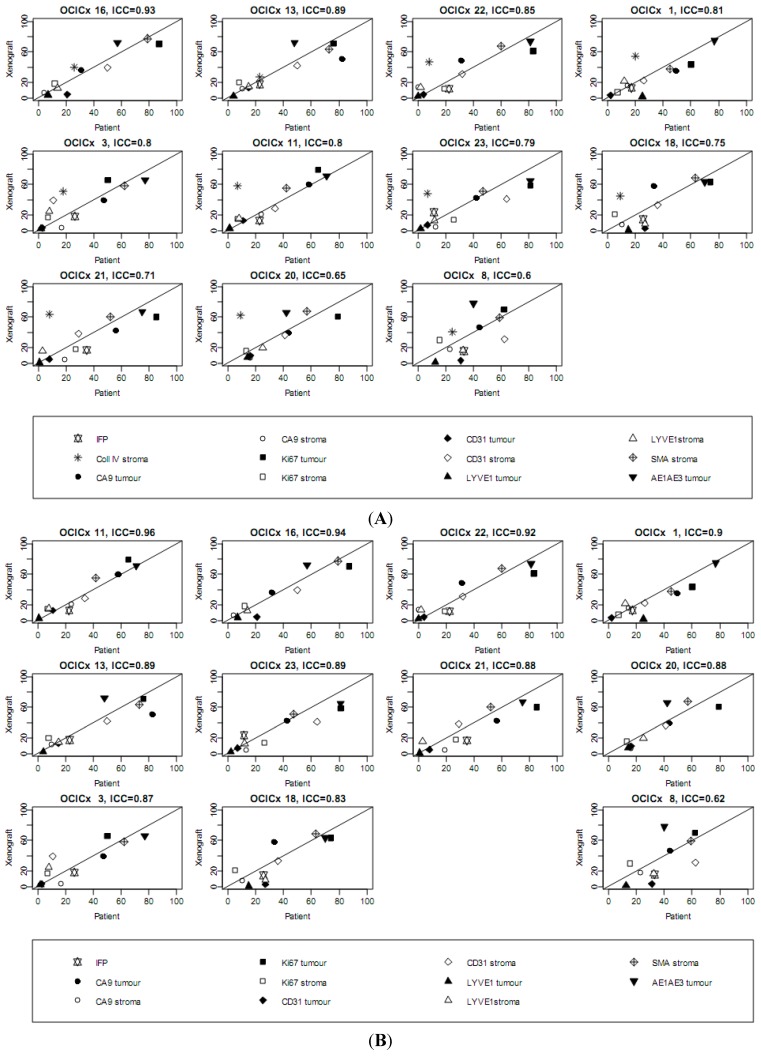
The relationship between the passage 3 xenograft for all models and its matching biopsy. Plots for IFP and tumor and stroma measures for hypoxia, blood and lymphatic vessels, proliferation and smooth muscle actin. Lines plotted are the (0,1) line of perfect concordance. Collagen IV is shown in the full data set in (**A**) and excluded as an outlier in (**B**). Values are listed in Supplementary Table 2.

To make an overall comparison of the concordance of the measurements in the biopsies and xenografts, we plotted all the data measured in the xenografts against that measured in the original biopsy for each of the 11 xenograft models (up to OCICx23), as shown in Figure 8A. The actual values are presented in Supplementary Table 3. To assess the reproducibility of the patient measurements in the relevant xenografts the Intraclass Correlation Coefficient (ICC) was calculated and the data are plotted in the order of the ICC. Notably in the Figure 8A the collagen IV staining levels (in stroma) generally show a poor correlation, consequently we also plotted the data excluding the collagen IV data (Figure 8B), again ordered according to the ICC values. The majority of models (10 of 11) show good concordance (ICC ≥ 0.83) with OCICx 11, 16, 22 and 1 as the first 4 models with strong positive correlations for all markers. One of the xenograft model (OCICx 8) associated poorly, regardless of whether the collagen IV measurements are included or not (ICC = 0.62 excluding Collagen IV and ICC = 0.6 for the entire data set, respectively).

## 4. Discussion

The aim of this present study was to characterize the tumor microenvironment of primary cervix xenografts, established and propagated directly from patient tumors. Our goal was to develop well-characterized and representative models of human tumors that recapitulate the microenvironmental complexity of clinical cervical cancer. To our knowledge there have been no other studies of this nature demonstrating the differences in the tumor microenvironment between early xenograft passages. We report the establishment of a unique set of serially transplantable, orthotopic, patient-derived cervical cancer xenograft models that, at least within the range of passage numbers examined, show a relatively stable retention of the original tumor characteristics, but demonstrate a range of features faithfully reflecting the tumor heterogeneity encountered in patients. This is essential for the validity of the xenograft model and the results which will be obtained from them using novel therapeutics [49]. Direct orthotopic implantation of the tumors may preserve the ability of cells to interact with supporting cells such as the stroma within the microenvironment; features that are lost in the in vitro setting. However, one drawback of any tumor grafting model based on core biopsies is that small acquired samples may not always be representative of an entire patient tumor [50].

There are a limited number of studies reporting on the development of patient-derived xenograft models through serial orthotopic implantation. The latency for tumor “take” is variable among tumour histology, location of implantation, and recipient strain [51]. Studies in patient-derived xenografts of pancreatic, lung, ovarian and prostate carcinomas demonstrate engraftment rates ranging between 40–95% with serial passages resembling the original biopsy histologically [52,53]. See Supplementary Table 2 for summary. Recently DeRose *et al*. [54] reported on the establishment of serially transplantable, orthotopic, breast tumor grafts that retain the characteristics of the original tumor (based on histology and genetic profiling). The tumor architecture was maintained through several passages in mice including its supportive stroma. Furthermore, Jin *et al*. [55] showed that early passage of the patient-derived tumor tissue of primary colon carcinoma, lymphatic and hepatic metastases revealed a high level of similarity with the original clinical samples with respect to histology, immunohistochemistry and gene expression. Thus it is clear that patient derived xenograft models histopathological features and molecular signatures that are similar to the original tumors in various contexts. There are limited studies investigating orthotopic *vs*. subcutaneous xenografts comparisons originating from patient tissue samples. However Pocard *et al*. [56] does in fact show, in colorectal carcinomas, that orthotopic xenografts resemble the original tissue sample better then subcutaneous tumors.

However, there has been limited detailed analyses about the stromal and microenvironmental components of these primary xenograft models particularly in regards to changes during passaging. In our primary cervix xenograft model an engraftment rate of approximately 48% was found. This rate was not correlated to HPV status or patient clinico-pathological features nor to the measurements of hypoxia (HP5) or IFP in the primary (patient) tumor, although it should be recognized that this may reflect the small sample size of the biopsy population. Despite the lack of correlation with clinic-pathologic features we did observe the development of lymphatic metastases in many of the models and observed that the number of metastases increased with passage number in the four models analysed for passage number changes. Consistent with other studies, as noted above, we observed that the OCICx xenografts generally track with the patient biopsy in terms of tumor morphology and stromal elements. The strong positive correlation between the pathologist scoring of the tumor sections for epithelial and stromal components and the automated Genie system (Figure 3) confirms the value of the automated approach to allow quantitative analysis of specific markers across whole tumor sections.

The analysis of differences in the various measurements and markers during the passaging of the xenografts suggests that hypoxia levels increase with passage number (generation) in the tumors. In parallel with the increase in EF5 and CA-9, particularly in the epithelial component, there is an increase in the proliferation marker, Ki-67, in the (oxic) epithelial component, which may increase oxygen demand. The increased levels of CD-31 staining (endothelial cells) in the epithelial and stromal components are consistent with such an increased demand but the increased level of hypoxia suggests that this does not result in a sufficient increase in supply of oxygen. Presumably the levels of hypoxia in the xenografts must plateau and stabilize at later passages. A recent study assessing levels of hypoxia in pancreatic cancer xenografts (at later passages than used here) using EF5 staining and analysis of the whole tumor with flow cytometry, reported that, for xenografts from different primaries, there were consistently lower or higher values of hypoxia [57]. This study also observed higher Ki-67 levels in tumors with greater levels of hypoxia.

It can be noted that the EF-5 staining levels in both the epithelial and stromal compartments tend to be lower than those for CA-9. Although this may reflect factors other than hypoxia than can modulate CA-9 expression it is also consistent with findings that lower levels of oxygen are required to activate EF-5 binding than are required to stabilize HIF-1 and stimulate CA-9 production [58]. This highlights an often unappreciated difference between these hypoxia markers. Also the levels of hypoxia and Ki-67 staining are higher and increase faster in the epithelial component than the stromal component, whilst the opposite is true for the CD-31 expression, consistent with most of the vasculature remaining within the stromal compartment and limited invasion of vessels into the tumor component. LYVE-1 staining (primarily reflecting lymphatics) shows a similar pattern to the CD-31 staining. It is particularly low in the epithelial component consistent with low levels of lymphatics in tumors. Similarly, Ki-67 levels are much lower in the stromal than the epithelial components. However, measurements within a specific tumor are subject to heterogeneity inherent within that model (*i.e*., in tumors from different animals but from the same model) (Figure 7). Mean values from sections derived from multiple individual tumors were felt to provide a better global perspective of a particular xenograft model (derived from a specific patient). Unfortunately this does not translate to the comparison with the primary patient biopsy, which being a core biopsy only represents one region of the primary tumor and cannot encompass the heterogeneity inherent in that patient’s cancer.

The small amount of tissue available for analysis of the biopsies likely explains why the individual markers showed limited correlation between the biopsy and their respective xenografts. Nevertheless when all of the markers were combined the intraclass correlation coefficient analysis (Figure 8) showed that overall there was good correlation between the patient biopsy and the measurements of the markers in all the xenografts at passage three with the exception of collagen IV. Reasons why collagen IV is an outlier in most of the OCICx models are unclear but we note that different antibodies were used for the xenografts and the biopsies, so in the case of the xenografts any residual human stromal collagen IV would not have been detected. For all the other IHC markers the other antibodies recognised both the human and murine markers. Excluding collagen IV from the ICC analysis showed a good correlation for other markers for all but one of the eleven xenograft models analysed. While it is possible that heterogeneity explains the failure of this model to show a good correlation the data raises a caution that not all xenografts may necessarily be good models of the tumour from which they are derived, even at very early passage. Genetic analysis of the xenograft models is currently planned and it will be of interest to determine if the degree of correlation between the biopsy and xenograft models parallels that observed with the current markers. Based on the available data in the literature [51] for such comparisons we anticipate that all the models will show a high correlation.

Currently we are further exploring the tumor microenvironment factors such as stromal components and hypoxia in these xenografts through a multimodality imaging study (manuscript in preparation). These imaging studies will help to address some of the issues associated with heterogeneity both within the xenografts and between xenograft and primary, which, as noted above, is a concern because the primary tumor is represented in the current study by a single biopsy. Inevitably imaging measurements are at a much lower resolution than is possible with tissue sections.

## 5. Conclusions

Overall our results demonstrate that early passage xenografts established from clinical cervix cancer biopsies, capture and reflect the general histopathological features of the primary tumor not only in terms of epithelial *vs.* stromal elements but other tumor microenvironmental elements (hypoxia, IFP, vasculature). However, detailed IHC analyses suggest significant increases in hypoxia and proliferation in the epithelial component during serial passaging. This was associated with an increase in the number of lymphatic metastases with passage number suggesting a possible increase in malignancy. An important caveat is that the interpretation of our studies must be cautious because of the known issues of heterogeneity within the tumor but the data has significant implications for the choice of suitable xenograft models for therapeutic testing of drugs. Understanding the importance of the stroma with the tumor microenvironment in our model of the primary cervix xenografts remains a work-in-progress and further analysis is warranted.

## Supplementary Materials

**Supplementary Table 1:** Patient-derived cervix xenografts (OCICx). A list of 16 OCICx xenografts generated from cervix patient biopsies with details of the clinical background from the patient and tumor grade (SCC = squamous, AD = adenocarcinoma). The xenografts are passaged up to 5 generations and the number of orthotopic tumors in each passage is indicated. NA indicates that tumors have not yet reached up to this passage. The analysis has been done on passage 3 tumors and on multiple passages on OCICx 1, 3, 8, 16.

OCICx xenografts																		
	Biopsy	1	3	8	11	13	15	16	18	20	21	22	23	26		28	29	30
Passage		IIB; SCC	IIA; SCC	IIB; AD	IB; SCC	IIA; AD	IIA; AD	IIIB; SCC	IIB; SCC	IIIB; SCC	IIIB; SCC	IIB; * other	IIB; SCC	IIB; clear cell		IIB; SCC	IB; SCC	IIB; SCC
1		2	6	2	1	2	1	5	3	2	3	3	2		4	1	4	1
2		8	11	6	2	2	1	12	2	3	5	2	3		1	4	3	NA
3		21	9	11	3	3	2	12	4	7	4	4	4		NA	NA	NA	NA
4		25	13	8	4	1	4	12	6	7	5	1	3		NA	NA	NA	NA
5		14	4	7	4	2	5	14	7	3	7	3	3		NA	NA	NA	NA

* Other: mucinous.

**Supplementary Table 2:** Summary of various patient derived tumor models showing engraftment rate (%) and site of tumor growth.

Tumor type	Engraftment site	Take rate %	References
**Lung**	subcutaneous	40	John *et al.* [34]
**Breast**	mammary fat pad	37	DeRose *et al.* [54]
**Ovary**	subrenal capsule	95	Lee *et al.* [52]
**Pancreas**	subcutaneous	80	Rubio-Viqueira *et al.* [53]
**Prostate**	subrenal capsule	56	Priolo *et al.* [37]
**Colon**	subcutaneous	60	Jin *et al.* [55]
**Renal**	kidney capsule	95	Grisanzio *et al.* [39]
**Osteosarcoma**	subcutaneous	20	Mayordomo *et al.* [38]

**Supplementary Table 3:** Percent labelling for IHC markers for passage 3 xenografts (mean ±SE) and matching biopsies (single values) measured in stroma (**A**) and tumor (**B**).

Marker	IFP		CollIV stroma		CA9 stroma		CD31 stroma		Ki67 stroma		LYVE1 stroma		SMA stroma	
OCICx	*Pt*	*Xeno*	*Pt*	*Xeno*	*Pt*	*Xeno*	*Pt*	*Xeno*	*Pt*	*Xeno*	*Pt*	*Xeno*	*Pt*	*Xeno*
**1**	17.3	13.3 (10.9–15.6)	20	54.7 (52.9–56.4)	14	15.3 (13.5–17.1)	26	24.3 (22.7–25.9)	7	7 (4.6–9.3)	12.0	24.8 (21.6–28.1)	45.0	37.8 (36.7–38.9)
**3**	26.5	16.1 (12.5–19.6)	18	51 (43–59)	17	3.7 (2.6–4.8)	11	41.7 (37.9–45.5)	7	16.4 (14.9–17.9)	8.0	24.2 (22.2–26.3)	62.0	58.5 (50.9–66.1)
**8**	33.2	14 (9.4–18.7)	25	40.8 (38.1–43.4)	23	17.6 (14.5–20.8)	62.5	32.5 (27.5–37.5)	16	30.4 (18.6–42.2)	32.5	13.6 (7.5–19.8)	59.0	59.2 (56.4–62.1)
**11**	22.7	12.9 (7.4–18.4)	7	58 (46.1–69.9)	24	21 (6.2–35.8)	34	28.7 (24.7–32.6)	7	15.3 (12.5–18.2)	8.0	16 (11.1–20.9)	42.0	55.7 (49.4–61.9)
**13**	23	17.7 (14.3–21)	23	27.3 (13–41.7)	10	11.7 (11–12.3)	50	42.3 (33.3–51.4)	8	20.3 (11.3–29.4)	15.0	15 (12.7–17.3)	73.0	64 (56.6–71.4)
**15**	NA	9 (7.6–10.4)	27	63 (59.1–66.9)	0	12.5 (7.6–17.4)	54	30 (16.3–43.7)	3	13.5 (12.5–14.5)	16.0	10 (10–10)	59.0	53 (27.5–78.5)
**16**	NA	15.5 (13.1–17.8)	26	40.2 (31–49.5)	4	6.4 (4.2–8.6)	50	39.6 (37.1–42)	12	18.5 (15.8–21.3)	14.0	12.8 (9.9–15.8)	79.0	77.5 (71.9–83.1)
**18**	25.5	14.5 (10.2–18.8)	9	45 (29.5–60.5)	10	8 (−0.9 to 16.9)	36	33.2 (28.5–38)	5	21 (13.2–28.8)	27.0	8.2 (3–13.5)	63.0	68.5 (62.9–74.1)
**20**	NA	19.5 (16.3–22.7)	9	62.3 (60–64.5)	16	6 (0.6–11.4)	41	37.8 (32.4–43.1)	13	16.1 (11.6–20.6)	25.0	19 (15.4–22.6)	57.0	67.5 (61.9–73.1)
**21**	34.9	17.8 (15.3–20.3)	8	61 (55.8–66.2)	19	6.7 (3.1–10.2)	29	35 (26.2–43.8)	27	19.8 (13–26.7)	3.0	15.8 (13.1–18.6)	52.0	60.2 (56.6–63.9)
**22**	22.4	11.8 (9.9–13.6)	8	46.8 (41–52.5)	0	13.8 (9.1–18.4)	32	31.2 (26.6–35.9)	19	12.2 (10.4–14.1)	2.0	14 (9.2–18.8)	60.0	68 (64.5–71.5)
**23**	11.6	23.9 (13.5–34.4)	7	48.3 (39.5–57)	13	5.3 (1.7–8.8)	64	41.2 (34.8–47.7)	26	13.8 (9.1–18.4)	12.0	13.2 (8.8–17.7)	47.0	51.2 (42–60.5)
**(A) **														
**Marker**	**CA9 tumor**			**CD31 tumor**			**Ki67 tumor**			**LYVE1 tumor**			**AE1AE3 tumor**	
**OCICx**	Pt	Xeno		Pt	Xeno		Pt	Xeno		Pt	Xeno		Pt	Xeno
**1**	49	34.8 (32.2–37.3)		2	3.4 (2.8–4)		60	43.5 (41–46.1)		25	1.9 (1.1–2.6)		77	74.7 (74.1–75.2)
**3**	47	39.8 (36.1–43.6)		3	3.9 (2.5–5.4)		50	65.2 (62.3–68)		2	2.2 (1.6–2.9)		77	66.2 (63.3–69.2)
**8**	44	47.6 (40.5–54.8)		31	3.4 (2.4–4.4)		62	71.5 (63.5–79.5)		13	1.1 (0.7–1.4)		40	78 (74.1–81.9)
**11**	58	60.3 (55.8–64.9)		11	13 (−1.4 to 27.4)		65	79 (75.6–82.4)		1	2.7 (2–3.3)		71	71.3 (62.7–80)
**13**	82	51.7 (43–60.3)		15	13.3 (7.8–18.9)		76	71 (61.1–80.9)		4	2 (−1 to 5)		48	72 (60.5–83.5)
**15**	50	48.5 (41.6–55.4)		32	9.5 (8.5–10.5)		57	73.5 (72.5–74.5)		4	1 (1–1)		22	74 (68.1–79.9)
**16**	31	36.5 (31.8–41.2)		21	4.5 (2.8–6.2)		87	70.5 (65.5–75.4)		7	3.8 (2–5.5)		57	72 (68.1–75.9)
**18**	33	58.5 (52.6–64.4)		27	2.8 (1.1–4.4)		74	63 (50.3–75.7)		15	0.8 (−0.2 to 1.7)		70	63.3 (56.1–70.6)
**20**	43	39 (28.8–49.2)		16	8.8 (3.8–13.7)		79	62.8 (54–71.5)		14	7.8 (5.5–10)		42	66 (63.2–68.8)
**21**	56	48.7 (38.2–59.1)		8	4.2 (2.5–5.8)		85	62.7 (56.5–68.8)		1	1.2 (0–2.3)		75	66.8 (60–73.5)
**22**	31	48.8 (36.5–61)		4	4.2 (3.3–5.2)		83	61 (46.7–75.3)		0	2 (0.6–3.4)		81	73.8 (64.1–83.4)
**23**	42	43.2 (35.2–51.3)		7	7 (3.5–10.5)		81	58.2 (51.3–65.2)		2	2.2 (0.1–4.4)		81	65 (59.5–70.5)
**(B) **														

## References

[1] Hu J., Bianchi F., Ferguson M., Cesario A., Margaritora S., Granone P., Goldstraw P., Tetlow M., Ratcliffe C., Nicholson A.G. (2005). Gene expression signature for angiogenic and nonangiogenic non-small-cell lung cancer. Oncogene.

[2] Zagouri F., Sergentanis T.N., Chrysikos D., Filipits M., Bartsch R. (2012). Molecularly targeted therapies in cervical cancer. A systematic review. Gynecol. Oncol..

[3] Anttila A., von Karsa L., Aasmaa A., Fender M., Patnick J., Rebolj M., Nicula F., Vass L., Valerianova Z., Voti L. (2009). Cervical cancer screening policies and coverage in Europe. Eur. J. Cancer.

[4] Parkin D.M., Bray F., Ferlay J., Pisani P. (2005). Global cancer statistics, 2002. CA Cancer J. Clin..

[5] Scholl S.M., Kenter G., Kurzeder C., Beuzeboc P. (2011). Pathway profiling and rational trial design for studies in advanced stage cervical carcinoma: A review and a perspective. ISRN Oncol..

[6] Ferlay J., Shin H.R., Bray F., Forman D., Mathers C., Parkin D.M. (2010). Estimates of worldwide burden of cancer in 2008: GLOBOCAN 2008. Int. J. Cancer.

[7] Gius D., Funk M.C., Chuang E.Y., Feng S., Huettner P.C., Nguyen L., Bradbury C.M., Mishra M., Gao S., Buttin B.M. (2007). Profiling microdissected epithelium and stroma to model genomic signatures for cervical carcinogenesis accommodating for covariates. Cancer Res..

[8] Cuzick J., Arbyn M., Sankaranarayanan R., Tsu V., Ronco G., Mayrand M.H., Dillner J., Meijer C.J. (2008). Overview of human papillomavirus-based and other novel options for cervical cancer screening in developed and developing countries. Vaccine.

[9] Tiltman A.J. (2005). The pathology of cervical tumours. Best Pract. Res. Clin. Obstet. Gynaecol..

[10] Fyles A.W., Milosevic M., Wong R., Kavanagh M.C., Pintilie M., Sun A., Chapman W., Levin W., Manchul L., Keane T.J. (1998). Oxygenation predicts radiation response and survival in patients with cervix cancer. Radiother. Oncol..

[11] Fyles A., Milosevic M., Hedley D., Pintilie M., Levin W., Manchul L., Hill R.P. (2002). Tumor hypoxia has independent predictor impact only in patients with node-negative cervix cancer. J. Clin. Oncol..

[12] Fyles A., Keane T.J., Barton M., Simm J. (1992). The effect of treatment duration in the local control of cervix cancer. Radiother. Oncol..

[13] Lu K.H., Burke T.W. (2000). Early cervical cancer. Curr. Treat. Options Oncol..

[14] Morris M., Eifel P.J., Lu J., Grigsby P.W., Levenback C., Stevens R.E., Rotman M., Gershenson D.M., Mutch D.G. (1999). Pelvic radiation with concurrent chemotherapy compared with pelvic and para-aortic radiation for high-risk cervical cancer. New Engl. J. Med..

[15] Rose P.G. (2002). Chemoradiotherapy for cervical cancer. Eur. J. Cancer.

[16] Tewari K.S., Monk B.J. (2009). Recent achievements and future developments in advanced and recurrent cervical cancer: Trials of the Gynecologic Oncology Group. Semin. Oncol..

[17] Kesic V. (2006). Management of cervical cancer. Eur. J. Surg. Oncol..

[18] Ungefroren H., Sebens S., Seidl D., Lehnert H., Hass R. (2011). Interaction of tumor cells with the microenvironment. Cell Commun. Signal..

[19] Lunt S.J., Kalliomaki T.M., Brown A., Yang V.X., Milosevic M., Hill R.P. (2008). Interstitial fluid pressure, vascularity and metastasis in ectopic, orthotopic and spontaneous tumours. BMC Cancer.

[20] Sleeman J.P., Christofori G., Fodde R., Collard J.G., Berx G., Decraene C., Ruegg C. (2012). Concepts of metastasis in flux: The stromal progression model. Semin. Cancer Biol..

[21] Li H., Fan X., Houghton J. (2007). Tumor microenvironment: The role of the tumor stroma in cancer. J. Cell. Biochem..

[22] Noman M.Z., Messai Y., Carre T., Akalay I., Meron M., Janji B., Hasmim M., Chouaib S. (2011). Microenvironmental hypoxia orchestrating the cell stroma cross talk, tumor progression and antitumor response. Crit. Rev. Immunol..

[23] Plzak J., Lacina L., Chovanec M., Dvorankova B., Szabo P., Cada Z., Smetana K. (2010). Epithelial-stromal interaction in squamous cell epithelium-derived tumors: An important new player in the control of tumor biological properties. Anticancer Res..

[24] Mueller M.M., Fusenig N.E. (2004). Friends or foes—Bipolar effects of the tumour stroma in cancer. Nat. Rev. Cancer.

[25] Nordsmark M., Loncaster J., Aquino-Parsons C., Chou S.C., Ladekarl M., Havsteen H., Lindegaard J.C., Davidson S.E., Varia M., West C. (2003). Measurements of hypoxia using pimonidazole and polarographic oxygen-sensitive electrodes in human cervix carcinomas. Radiother. Oncol..

[26] Hockel M., Schlenger K., Hockel S., Vaupel P. (1999). Hypoxic cervical cancers with low apoptotic index are highly aggressive. Cancer Res..

[27] Fyles A., Milosevic M., Pintilie M., Syed A., Levin W., Manchul L., Hill R.P. (2006). Long-term performance of interstial fluid pressure and hypoxia as prognostic factors in cervix cancer. Radiother. Oncol..

[28] Milosevic M., Fyles A., Hedley D., Pintilie M., Levin W., Manchul L., Hill R. (2001). Interstitial fluid pressure predicts survival in patients with cervix cancer independent of clinical prognostic factors and tumor oxygen measurements. Cancer Res..

[29] Ellingsen C., Hompland T., Mathiesen B., Rofstad E.K. (2012). Microenvironment-associated lymph node metastasis of human cervical carcinoma xenografts. Acta Oncol..

[30] Bernhard E.J. (2011). Interventions that induce modifications in the tumor microenvironment. Cancer Radiother..

[31] Milosevic M., Fyles A., Hedley D., Hill R. (2004). The human tumor microenvironment: Invasive (needle) measurement of oxygen and interstitial fluid pressure. Semin. Radiat. Oncol..

[32] Jain R.K., Tong R.T., Munn L.L. (2007). Effect of vascular normalization by antiangiogenic therapy on interstitial hypertension, peritumor edema, and lymphatic metastasis: Insights from a mathematical model. Cancer Res..

[33] Rofstad E.K., Ruud E.B., Mathiesen B., Galappathi K. (2010). Associations between radiocurability and interstitial fluid pressure in human tumor xenografts without hypoxic tissue. Clin. Cancer Res..

[34] John T., Kohler D., Pintilie M., Yanagawa N., Pham N.A., Li M., Panchal D., Hui F., Meng F., Shepherd F.A. (2011). The ability to form primary tumor xenografts is predictive of increased risk of disease recurrence in early-stage non-small cell lung cancer. Clin. Cancer Res..

[35] Jin K., Teng L., Shen Y., He K., Xu Z., Li G. (2010). Patient-derived human tumour tissue xenografts in immunodeficient mice: A systematic review. Clin. Transl. Oncol..

[36] Frapolli R., Tamborini E., Virdis E., Bello E., Tarantino E., Marchini S., Grosso F., Sanfilippo R., Gronchi A., Tercero J.C. (2010). Novel models of myxoid liposarcoma xenografts mimicking the biological and pharmacologic features of human tumors. Clin. Cancer Res..

[37] Priolo C., Agostini M., Vena N., Ligon A.H., Fiorentino M., Shin E., Farsetti A., Pontecorvi A., Sicinska E., Loda M. (2010). Establishment and genomic characterization of mouse xenografts of human primary prostate tumors. Am. J. Pathol..

[38] Mayordomo E., Machado I., Giner F., Kresse S.H., Myklebost O., Carda C., Navarro S., Llombart-Bosch A. (2010). A tissue microarray study of osteosarcoma: Histopathologic and immunohistochemical validation of xenotransplanted tumors as preclinical models. Appl. Immunohistochem. Mol. Morphol..

[39] Grisanzio C., Seeley A., Chang M., Collins M., di Napoli A., Cheng S.C., Percy A., Beroukhim R., Signoretti S. (2011). Orthotopic xenografts of RCC retain histological, immunophenotypic and genetic features of tumours in patients. J. Pathol..

[40] Nemati F., Sastre-Garau X., Laurent C., Couturier J., Mariani P., Desjardins L., Piperno-Neumann S., Lantz O., Asselain B., Plancher C. (2010). Establishment and characterization of a panel of human uveal melanoma xenografts derived from primary and/or metastatic tumors. Clin. Cancer Res..

[41] Wang J., Daphu I., Pedersen P.H., Miletic H., Hovland R., Mork S., Bjerkvig R., Tiron C., McCormack E., Micklem D. (2011). A novel brain metastases model developed in immunodeficient rats closely mimics the growth of metastatic brain tumours in patients. Neuropathol. Appl. Neurobiol..

[42] Cairns R.A., Hill R.P. (2004). Acute hypoxia enhances spontaneous lymph node metastasis in an orthotopic murine model of human cervical carcinoma. Cancer Res..

[43] Chaudary N., Hedley D.W., Hill R.P. (2011). Orthotopic xenograft model of cervical cancer for studying microenvironmental effects on metastasis formation and response to drug treatment. Curr. Protoc. Pharmacol..

[44] Evans S.M., Hahn S.M., Magarelli D.P., Koch C.J. (2001). Hypoxic heterogeneity in human tumors: EF5 binding, vasculature, necrosis, and proliferation. Am. J. Clin. Oncol..

[45] Mayer A., Hockel M., Vaupel P. (2006). Endogenous hypoxia markers in locally advanced cancers of the uterine cervix: Reality or wishful thinking?. Strahlenther. Onkol..

[46] Hedley D., Pintilie M., Woo J., Morrison A., Birle D., Fyles A., Milosevic M., Hill R. (2003). Carbonic anhydrase IX expression, hypoxia, and prognosis in patients with uterine cervical carcinomas. Clin. Cancer Res..

[47] Ludwig J.A., Weinstein J.N. (2005). Biomarkers in cancer staging, prognosis and treatment selection. Nat. Rev..

[48] Iakovlev V.V., Pintilie M., Morrison A., Fyles A.W., Hill R.P., Hedley D.W. (2007). Effect of distributional heterogeneity on the analysis of tumor hypoxia based on carbonic anhydrase IX. Lab. Invest..

[49] Loukopoulos P., Kanetaka K., Takamura M., Shibata T., Sakamoto M., Hirohashi S. (2004). Orthotopic transplantation models of pancreatic adenocarcinoma derived from cell lines and primary tumors and displaying varying metastatic activity. Pancreas.

[50] Cutz J.C., Guan J., Bayani J., Yoshimoto M., Xue H., Sutcliffe M., English J., Flint J., LeRiche J., Yee J. (2006). Establishment in severe combined immunodeficiency mice of subrenal capsule xenografts and transplantable tumor lines from a variety of primary human lung cancers: Potential models for studying tumor progression-related changes. Clin. Cancer Res..

[51] Tentler J.J., Tan A.C., Weekes C.D., Jimeno A., Leong S., Pitts T.M., Arcaroli J.J., Messersmith W.A., Eckhardt S.G. (2012). Patient-derived tumour xenografts as models for oncology drug development. Nat. Rev. Clin. Oncol..

[52] Lee C.H., Xue H., Sutcliffe M., Gout P.W., Huntsman D.G., Miller D.M., Gilks C.B., Wang Y.Z. (2005). Establishment of subrenal capsule xenografts of primary human ovarian tumors in SCID mice: potential models. Gynecol. Oncol..

[53] Rubio-Viqueira B., Jimeno A., Cusatis G., Zhang X., Iacobuzio-Donahue C., Karikari C., Shi C., Danenberg K., Danenberg P.V., Kuramochi H. (2006). An *in vivo* platform for translational drug development in pancreatic cancer. Clin. Cancer Res..

[54] DeRose Y.S., Wang G., Lin Y.C., Bernard P.S., Buys S.S., Ebbert M.T., Factor R., Matsen C., Milash B.A., Nelson E. (2011). Tumor grafts derived from women with breast cancer authentically reflect tumor pathology, growth, metastasis and disease outcomes. Nat. Med..

[55] Jin K., Li G., Cui B., Zhang J., Lan H., Han N., Xie B., Cao F., He K., Wang H. (2011). Assessment of a novel VEGF targeted agent using patient-derived tumor tissue xenograft models of colon carcinoma with lymphatic and hepatic metastases. PloS One.

[56] Pocard M., Muleris M., Hamelin R., Salmon R.J., Dutrillaux B., Poupon M.F. (1998). Growth dependency of human colon cancer xenograft on organ environment is related with their original clinical stage. Anticancer Res..

[57] Chang Q., Jurisica I., Do T., Hedley D.W. (2011). Hypoxia predicts aggressive growth and spontaneous metastasis formation from orthotopically grown primary xenografts of human pancreatic cancer. Cancer Res..

[58] Wilson W.R., Hay M.P. (2011). Targeting hypoxia in cancer therapy. Nat. Rev..

